# The Effects of Plasma Homocysteine Level on the Risk of Three Major Psychiatric Disorders: A Mendelian Randomization Study

**DOI:** 10.3389/fpsyt.2022.841429

**Published:** 2022-03-21

**Authors:** Jing Yu, Ranran Xue, Qiuling Wang, Hao Yu, Xia Liu

**Affiliations:** ^1^Department of Psychiatry, Jining Medical University, Jining, China; ^2^Department of Psychiatry, Shandong Daizhuang Hospital, Jining, China

**Keywords:** homocysteine, schizophrenia, bipolar disorder, major depressive disorder, genome-wide association studies, Mendelian randomization

## Abstract

**Background:**

Higher homocysteine (Hcy) level has been suggested to be associated with major psychiatric disorders (MPDs), such as schizophrenia (SCZ), bipolar disorder (BD), and major depressive disorder (MDD). We investigated the causal relationships between plasma Hcy level and MPDs risks using the Mendelian randomization (MR) method.

**Methods:**

We selected 18 loci associated with plasma Hcy level from a large-scale genome-wide association study (GWAS) as genetic instruments. Genetic associations with SCZ, MDD, BD and BD subtypes (BD-I and BD-II) were extracted from several GWAS datasets from the Psychiatric Genomics Consortium. We used the Generalized Summary-data-based Mendelian Randomization (GSMR) method to estimate the associations of genetically predicted plasma Hcy levels with MPDs risks. We also performed inverse variance-weighted (IVW) analysis to verify the GSMR results and used MR-Egger regression and leave-one-out analysis to test the assumptions for a valid MR analysis.

**Results:**

Genetically predicted plasma Hcy levels were associated with risks of SCZ (odds ratio [OR] = 1.12, *P*_*GSMR*_ = 1.73 × 10^−3^) and BD-I (OR = 1.14, *P*_*IVW*_ = 5.23 × 10^−3^) after Bonferroni correction. These associations were statistically significant when using IVW analysis (SCZ: OR = 1.11, *P*_IVW_ = 2.74 × 10^−3^; BD-I: OR = 1.13, *P*_*IVW*_ = 9.44 × 10^−3^). Furthermore, no significant horizontal pleiotropy was found by sensitivity analysis, and leave-one-out analyses showed no specific SNP affected the overall estimate. However, genetically determined plasma Hcy levels were not causally associated with MDD, BD, or BD-II risks.

**Conclusion:**

Our results suggest that elevated plasma Hcy levels may increase the risk of SCZ or BD-I. Further randomized clinical trials are warranted to validate the MR findings in our study.

## Introduction

Major psychiatric disorders (MPDs), such as schizophrenia (SCZ), bipolar disorder (BD), and major depression (MDD), are the major contributors to long-term disability and the burden of global health (1, 2). However, the pathogenesis of MPDs remains poorly understood. Therefore, robust biomarkers toward the prevention and treatment are needed to reduce the burden of MPDs.

Clinical studies have consistently found that plasma homocysteine (Hcy) level was associated with risk of MPDs (3–7). Homocysteine is a non-proteogenic thiol amino acid, which the methionine synthase can catalyze into methionine (8). Several studies have shown increased plasma Hcy levels in patients with SCZ (6, 7), MDD (3), and BD (4, 5). Moreover, a randomized controlled trial found that plasma Hcy levels decreased significantly after risperidone treatment and PANSS scores decreased significantly in patients with SCZ (9), suggesting that high Hcy levels may be related to the pathogenesis of SCZ and clinical psychopathology. Besides, an increasing body of evidence suggests increased plasma Hcy levels are associated with an increased prevalence of more severe cognitive impairment in SCZ (10), MDD (11), and BD (12). Moreover, previous clinical trial suggested that serum Hcy levels were significantly decreased in patients with SCZ after risperidone treatment (9). However, few studies explored the relationships between Hcy level and the treatment of SCZ and BD. Meanwhile, no significant differences in Hcy levels were found when comparing patients with SCZ (13), MDD (14), and BD (15) to healthy controls. Thus, the causal relationship between Hcy level and MPDs risk remains unclear.

Mendelian randomization (MR) is a genetic epidemiological method for investigating putative causal effects of a risk factor (or exposure) on an outcome by utilizing genetic variants as instrumental variables (16, 17). In MR analysis, the random segregation of alleles can independently divide participants into exposure and control groups (16, 17). At the same time, potential confounding variables can be equally distributed between the two groups. As such, MR analysis can ingeniously minimize factors such as confounding and reverse causation in epidemiological research (18).

Meanwhile, the MR method can save time and cost based on genetic instrumental variables extracted from GWAS summary data (19). Given these advantages, the MR method has been widely used to assess causal associations between risk factors and complex diseases. Up to now, genome-wide association studies (GWAS) have identified hundreds of risk loci for plasma Hcy level (20) and MPDs (21–23), providing new opportunities to investigate the relationships between them.

Herein, leveraging large-scale GWAS summary data, we performed a two-sample MR analysis to examine the effects of plasma Hcy level on the risk of MPDs. We used genetic variants associated with plasma Hcy level as instrumental variables to improve inference of the impact of the Hcy on the risk of MPDs.

## Materials and Methods

### Study Design

**Figure 1:**
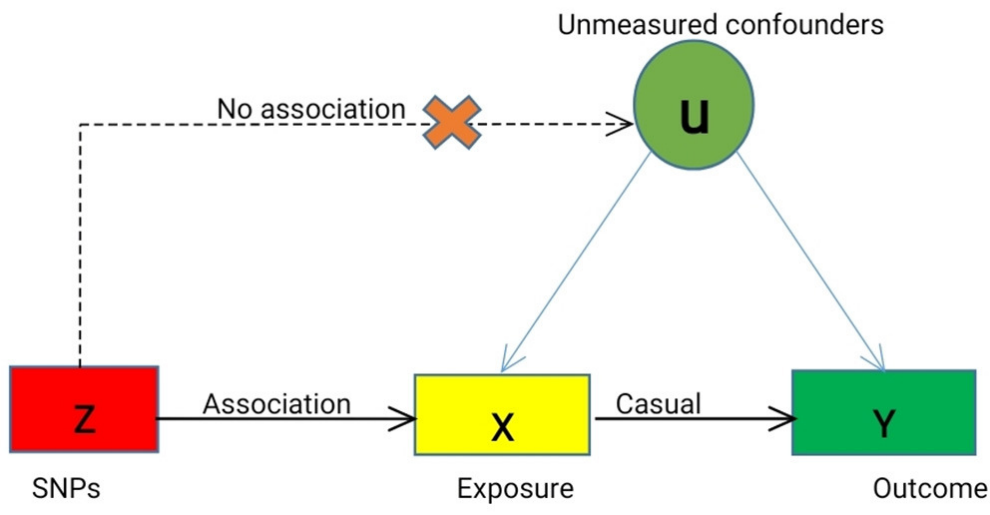
Mendelian Randomization Model. We conducted MR analyses based on the following assumptions, (a) the instrumental variables (IVs) for each of the Hcy level (exposure) are associated with the risk of MPDs (outcome), (b) the IVs are not associated with any confounders (U), and (c) the IVs are associated with MPDs only through Hcy level, not through other causal pathway. Solid lines are theorized to exist; dashed lines are theorized to be non-significant according to MR assumptions. Causal directed acyclic graph illustrating the instrumental variable assumptions for the instrumental variable, exposure X, outcome Y, and the set of variables (U) that confound the association between X and Y.

We designed a two-sample MR study to investigate the causal effect of plasma Hcy level on the risk of MPDs, including SCZ, MDD, and BD. We selected the single nucleotide polymorphisms (SNPs) as instrumental variables (IVs) for plasma Hcy level based on the following three basic assumptions (Figure 1): (1) the selected SNPs should be significantly associated with plasma Hcy level; (2) SNPs must be independent of confounders; (3) SNPs were only associated with the risk of MPDs by plasma Hcy levels.

### Data Sources

We obtained the genetic variants associated with plasma Hcy level from a large-scale GWAS meta-analysis, with up to 44,147 individuals of European ancestry (20). We selected SNPs associated with plasma Hcy levels at the genome-wide significance threshold (*P* < 5 × 10^−8^) from the plasma Hcy GWAS. We then extracted the associations between IVs and MPDs from GWAS datasets of the Psychiatric Genomics Consortium (PGC) among individuals of European ancestry. Genetic associations for SCZ based on a large-scale PGC3 meta-analysis included 67,390 cases and 94,015 controls of European ancestry (24). Genetic associations with MDD were also obtained from a GWAS based on 16,823 cases and 25,632 controls of European descent (25). The associations between the genetic instrument and BD for common variants (minor allele frequency [MAF] >1%) came from GWAS of BD from the Psychiatric Genomics Consortium (PGC) among individuals of European ancestry. Summary statistics for BD were obtained from a recent GWAS meta-analysis including 41,917 cases and 371,549 controls from the BD working group of the PGC (26). For BD subtypes, BD-I GWAS meta-analyses consisted of 25,060 cases and 449,978 controls, and BD-II GWAS included 6,781 cases and 364,075 controls. The basic characteristics of GWAS samples are listed in Table 1. All the summary statistics of MPDs are available on the PGC website (https://www.med.unc.edu/pgc/results-and-downloads). Descriptions of exposure and outcome sources, such as the number of controls and cases, population structure, and dataset source, are presented in Table 1.

**Table 1:** Description of GWAS summary statistics used for each phenotype.

**Phenotype**	**Sample size**	**Population**	**Consortium/PMID**	**Significant loci^a^**
SCZ	67,390 cases and 94,015 controls	European	PGC	270
MDD	16,823 cases and 25,632 controls	European	PGC	44
BD	41,917 cases and 371,549 controls	European	PGC	64
BD-I	25,060 cases and 449,978 controls	European	PGC	44
BD-II	6,781 cases and 364,075 controls	European	PGC	1
Hcy	44,147 individuals	European	23824729	18

a *The number of genome wide significant (GWS) loci for each phenotype. We extracted genetic variants with genome-wide significant (P < 5 × 10^−8^) associations for plasma homocysteine level. Then, we extracted the associations between genetic instrument and SCZ (24), MDD (22), and BD (23) from the GWAS data of Psychiatric Genomics Consortium (PGC, http://www.med.unc.edu/pgc/)*.

### Mendelian Randomization Analysis

To examine the causal relationship between Hcy and MPDs, we analyzed the direction of causal effects using Generalized Summary-data based on Mendelian Randomization (GSMR; http://cnsgenomics.com/software/gsmr/) (27). For the genetic variants associated with plasma Hcy level, we selected linkage disequilibrium (LD)-independent lead SNPs (*r*^2^ < 0.01 and windows: 1,000 kb) with a genome-wide significant level (*P* < 5 × 10^−8^) as instrumental variables in the GSMR analyses (27). Then, we performed the HEIDI-outlier test implemented in GSMR software to test for horizontal pleiotropy (*P*_*HEIDI*_ < 0.01) and remove SNPs with pleiotropic effects on plasma Hcy level and MPDs (27). This study included analyses of 3 MPDs and 2 BD subtypes. A Bonferroni corrected threshold of *P* = 0.01 was considered to be significant. To complement the GSMR analysis, we also conducted the MR analyses using the inverse-variance-weighted (IVW) regression method, implemented via the 'TwoSampleMR' R package (28, 29). We applied the IVW method to examine the effects of plasma homocysteine level on the risks of MPDs (30), and compare whether the effect size estimates from the IVW regression method were consistent with those calculated using the GSMR method. The inverse-variance weighted (IVW) method estimates the causal effect by combining the casual estimates of each SNP in a fixed-effect meta-analysis model (30). To further investigate horizontal pleiotropy, we used the “TwoSampleMR” package to perform the MR-Egger regression to detect and correct horizontal pleiotropy (28, 29, 31). The leave-one-out sensitivity analyses were performed to determine whether the overall estimate was disproportionately affected by a specific SNP (28, 29).

## Results

We obtained 18 SNPs associated with plasma Hcy levels at a genome-wide significance level (*P* < 5 × 10^−8^; Supplementary Table 1). Among these SNPs, 5 (rs12134663, rs12921383, rs7422339, rs2851391, and rs957140) were removed due to high LD with other genetic variants. Then, the remaining 13 SNPs were used as IVs in our MR analysis. We listed the LD correlation matrix for these 13 SNPs in Supplementary Table 2. The characteristics of these SNPs and their associations with plasma Hcy level and MPDs are shown in Supplementary Tables 3–7.

### The Causal Relationship Between Plasma Hcy Levels and SCZ

**Figure 2:**
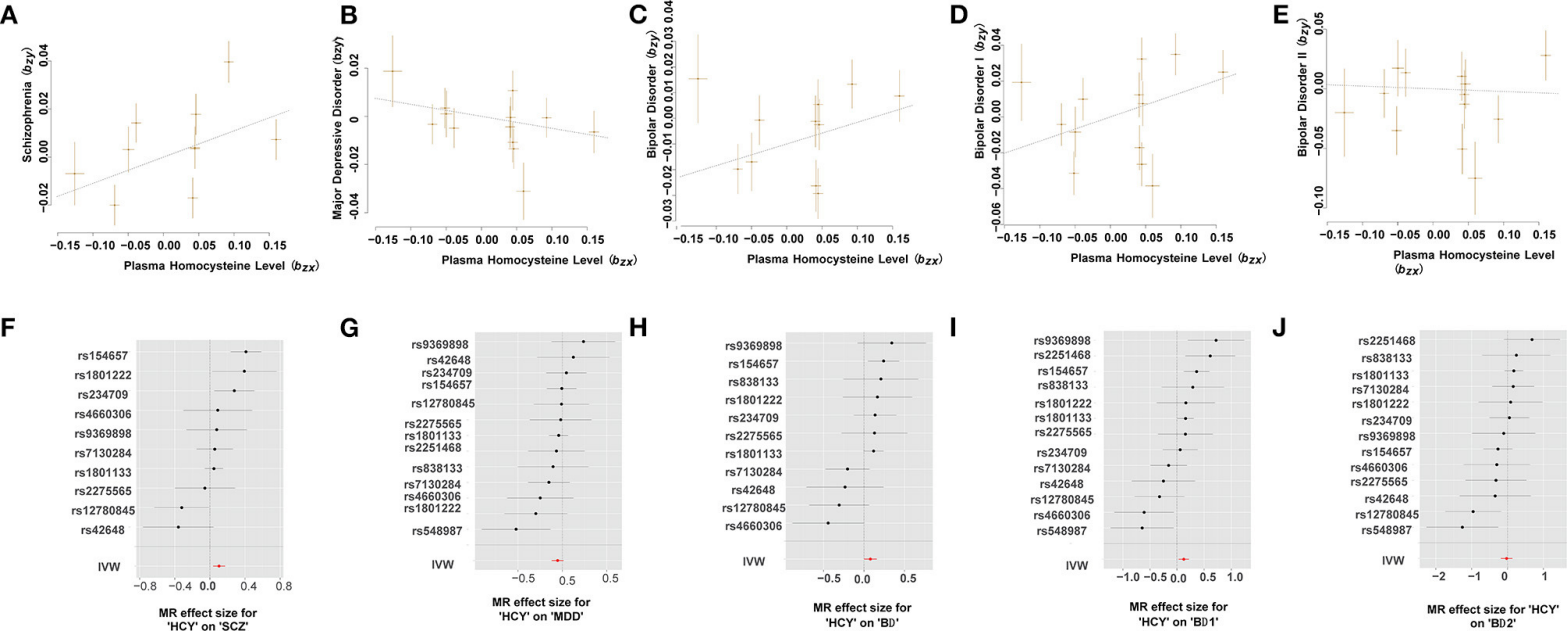
Mendelian randomization plots for relationship of Hcy level with MPDs. **(A–E)** Scatter plot of SNP potential effects on Hcy on SCZ **(A)**, MDD **(B)**, BD **(C)**, BD-I **(D)**, and BD-II **(E)**, with the slope of each line corresponding to estimated MR effect per method. **(F–J)** Forest plot of individual and combined SNP MR-estimated effects sizes for relationship of Hcy level with SCZ **(F)**, MDD **(G)**, BD **(H)**, BD-I **(I)**, and BD-II **(J)**. Data are expressed as beta values with 95% CI. IVW indicates inverse varianceweighted method.

Using the HEIDI-outlier method, we removed 3 SNPs (rs2251468, rs548987, and rs838133; *P*_*HEIDI*_ < 0.01; Supplementary Table 3) showed pleiotropic effects on plasma Hcy levels and SCZ and significantly deviated from causal models. Then, using the 10 remaining genetic instruments, we performed GSMR analysis, and our results showed that plasma Hcy level had a risk effect on SCZ (OR = 1.12, se = 0.035, *P*_*GSMR*_ = 1.73 × 10^−3^; Figure 2 and Table 2). An ORvalue of 1.12 indicates that individuals whose plasma Hcy level is increased by one Standard Deviation will have an increased risk of SCZ by 1.12 times compared with the population prevalence. Additionally, the effect size estimates from the IVW regression method (OR = 1.110, se = 0.039, *P*_*IVW*_ = 2.74 × 10^−3^; Table 2) were consistent with those calculated using the GSMR method. Moreover, our MR-Egger analysis suggested no evidence of bias from horizontal pleiotropy (intercept = −0.004, se = 0.01, *P* = 0.71). The leave-one-out analysis showed that no specific SNP affected the overall estimate (Supplementary Figure 1).

**Table 2:** MR estimates of the causality between homocysteine level and three major psychiatric disorders.

**Outcome**	**Number**	**MR Methods**	**OR**	**SE**	***P***
SCZ	10	GSMR	1.115	0.035	1.73E-03
		IVW	1.110	0.039	2.74E-03
MDD	13	GSMR	0.951	0.034	0.139
		IVW	0.948	0.032	0.115
BD	11	GSMR	1.088	0.040	0.037
		IVW	1.081	0.041	0.054
BD-I	13	GSMR	1.144	0.048	5.23E-03
		IVW	1.133	0.048	9.44E-03
BD-II	13	GSMR	0.977	0.082	0.783
		IVW	0.977	0.082	0.773

*We totally obtained 18 SNPs associated with plasma Hcy levels at a genome-wide significance level (P < 5 × 10^−8^). Of them, 5 SNPs (rs12921383, rs1801133, rs2851391, and rs957140) were removed due to high LD with other genetic variants. Then, we removed SNPs with pleiotropic effects on Hcy level and MPDs by using the HEIDI-outlier test. The associations between plasma Hcy level and MPDs were evaluated using Psychiatric Genomics Consortium (PGC) datasets as genetic instruments. We investigate the causal effects of Hcy on risks of MPDs using Generalized Summary-data based Mendelian Randomization (GSMR) method, with inverse variance weighted (IVW) method. SCZ, schizophrenia; MDD, major depressive disorder; BD, bipolar disorder; BD-I; BD-II, OR, Odds ratio, SE, standard error; P, P-value*.

### The Causal Relationship Between Plasma Hcy Levels and MDD

Our GSMR results suggested no evidence of a causal effect of plasma Hcy levels on MDD (OR = 0.951, SE = 0.034, *P*_*GSMR*_ = 0.139; Figure 2 and Table 2). Moreover, the IVW analysis also suggested no evidence of an association between genetically predicted plasma Hcy level and MDD (OR = 0.948, SE = 0.032, *P*_*IVW*_ = 0.115; Table 2). The estimates from MR-Egger regression (intercept = −0.001, se = 0.01, *P* = 0.86) analyses indicate that no horizontal pleiotropy exists. We detected no specific SNP affected the MR estimate in leave-one-out analyses (Supplementary Figure 2).

### The Causal Relationship Between Plasma Hcy Levels and BD

Using the HEIDI test, we removed two SNPs (rs2251468 and rs548987; *P*_*HEIDI*_ < 0.01), showing pleiotropic effects on plasma Hcy level and BD risk. Then, we conducted GSMR analysis using 11 retained SNPs and identified suggestive significant risk effects of plasma Hcy level on BD (OR = 1.088, SE = 0.04, *P*_*GSMR*_ = 0.037; Figure 2 and Table 2). Our IVW results also suggested the same direction of effect size estimates (OR = 1.081, se = 0.041, *P*_*IVW*_ = 0.05; Table 2), consistent with the GSMR results. However, the results were not significant after the Bonferroni correction (*P* > 0.01).

Then, we further examined the associations between plasma Hcy level and two BD subtypes, including BD-I and BD-II. We identified a significant risk effect of plasma Hcy level against BD-I (OR = 1.144, SE = 0.048, *P*_*GSMR*_ = 5.23 × 10^−3^; Figure 2 and Table 2), surviving after Bonferroni correction (*P* < 0.01). However, we found no evidence for a causal relationship between plasma Hcy level against BD-II (OR = 0.977, SE = 0.082, *P*_*GSMR*_ = 0.783; Figure 2 and Table 2). In addition, our IVW analysis also showed that plasma Hcy levels have a significant positive effect on BD-I risk (OR = 1.133, SE = 0.048, *P*_*IVW*_ = 9.44 × 10^−3^; Table 2), and no association between Hcy and BD-II (OR = 0.977, SE = 0.082, *P*_*IVW*_ = 0.773; Table 2). The associations were consistent in the MR-Egger regression analysis (BD-I: intercept = −0.006, se = 0.014, *P* = 0.68; BD-II: intercept = −0.020, se = 0.017, *P* = 0.26). Based on the leave-one-out sensitivity analyses, there was no evidence of obvious associations between plasma Hcy level with BD and its subtypes (Supplementary Figures 3–5).

## Discussion

Using a two-sample MR approach, we found that a genetic predisposition to higher plasma Hcy levels was causally linked to an increased risk of SCZ and BD-I. However, we did not find evidence supporting causal associations between plasma Hcy levels and MDD or BD-II. Our finding corroborates with many previous prospective observational studies that found that high plasma Hcy levels increased the risk of SCZ and BD. Our findings draw attention to the potential mental health consequences of Hcy dysfunction and provide more robust scientific evidence for future treatment guidelines. To the best of our knowledge, this is the first Mendelian randomization study with a sufficient sample size to examine whether there is a causal effect for a linear association between plasma Hcy level and risk of MPDs.

### Comparison With Previous Studies

Reports suggest Hcy may cause neurotoxicity by directly or indirectly activating glutamate receptors (32, 33). Based on the fact that Hcy and its oxidative metabolite, homocysteic acid, are N-methyl-D-aspartic-acid (NMDA) receptor agonists (32, 33), Hyperhomocysteinemia may cause long-term activation of NMDA receptors and exert neurotoxic and vasculotoxic effects (34). However, the definitive mechanism of elevated plasma Hcy levels in patients with SCZ or BD is still unclear. Our study provides further evidence for the causal effects of plasma Hcy levels on SCZ or BD-I risk. The accumulated evidence substantiates increased Hcy plasma levels are associated with SCZ or BD risk. A recent MR analysis in the Japanese population identified that increased plasma Hcy level was significantly associated with the risk of SCZ (35). Several observational studies found that the prevalence of hyperhomocysteinemia in patients with SCZ or BD was significantly higher than in normal controls (5, 36). Additionally, a meta-analysis provided evidence that plasma Hcy level is elevated in individuals with BD compared with healthy controls (4). Furthermore, some studies found that that Hcy was positively associated with the severity of SCZ, including negative symptoms (37) and cognitive functions (38–40). However, previous observational and epidemiological studies may suffer from confounding factors and limited sample size, leading to fallacious findings (16). In this regard, the MR method can ingeniously reduce the weaknesses of traditional research, providing a complementary method regarding etiology. Our MR results indicate significantly higher plasma Hcy levels were associated with SCZ or BD-I risk, which is in line with previous studies. Additionally, for the analysis of the association between Hcy and BD, most of the observational studies did not focus on BD subtypes. Herein, we interestingly found an increased level of plasma Hcy was associated with risk of BD-I, not with BD-II, suggesting that the one-carbon metabolism may be promising for clinical classification of BD. Notably, further randomized controlled trials need to be designed to verify the causal relationship between plasma Hcy level and MPDs risks.

Our study found no evidence to substantiate the causal association of Hcy with MDD. Many previous studies found that increased plasma Hcy levels may be associated with an increased risk of MDD (41), which is inconsistent with our MR findings. Multiple factors may account for this inconsistency. For instance, the sample size of traditional observational studies was not significant enough. Moreover, due to the presence of unobserved confounders and the excessively large number of confounders in observational studies, regression methods may fail to provide unbiased estimates of the true association. Additionally, elevated plasma Hcy levels detected in observational studies were potentially influenced by MDD.

### Mechanisms of Association

Our findings on the effects of Hcy on MPDs are compatible with known biology. Hcy may cause neurotoxicity by directly or indirectly activating glutamate receptors (32, 33). Since Hcy and its oxidative metabolite, homocysteic acid, are NMDA receptor agonists, hyperhomocysteinemia may cause long-term activation of NMDA receptors and exert neurotoxic and vasculotoxic effects (34). However, the definitive mechanism of elevated Hcy levels in patients with SCZ or BD is still unclear. It is speculated that poor nutrition, tobacco consumption, alcohol, coffee and polymorphisms in the enzymes of Hcy metabolism can all contribute to elevated Hcy levels (42, 43). Therefore, more emphasis should be placed on one-carbon metabolism in patients with SCZ or BD to improve the current understanding of disease pathogenesis.

### Clinical Relevance of Findings

Our findings provide the basis for potential clinical applications of plasma Hcy levels as a therapeutic target for SCZ or BD prevention. A large number of clinical studies have found that vitamin B12 and folic acid supplementation can reduce the plasma level of Hcy. With reduced plasma Hcy levels, the disease severity and cognitive function of patients with SCZ can significantly be improved (44, 45). Meanwhile, mood stabilizers (e.g., valproate and lamotrigine) used in treating BD can interfere in the folate and Hcy metabolism pathway (46). Recently, clinical research found that the vitamin B12 levels were significantly higher in responders with BD, suggesting that vitamin B12 supplements might be beneficial to treating patients with BD (47). Accordingly, more randomized clinical trials are warranted to validate whether lowering Hcy levels could alleviate the clinical symptoms of patients with SCZ or BD.

### Limitations

Our study has several limitations. First, our MR study identified the causal relationship between Hcy and SCZ/BD-I risk. However, the number of genetic instruments used in our MR analysis was limited. Further studies using more SNPs that are associated with plasma Hcy levels are needed. Second, our findings should be interpreted with caution since we could not completely reduce the effects of potential pleiotropy, which can be a source of biased estimates. However, the MR-Egger intercept test detected no evidence of pleiotropic effect in our study, and similar results were identified in sensitivity analyses using the leave-one-out method. Moreover, the SCZ or BD risk might be affected by exposure to higher Hcy levels at a specific period. Our MR analysis investigated lifelong elevated Hcy levels on SCZ/BD. However, genome-wide associations between SNPs and Hcy levels from adults were used. These associations in the early stage of the disease can be examined in the future. Furthermore, the examined GWAS were primarily conducted in individuals of European ancestry. Hence these results cannot be generalized to all populations. Besides, a reverse analysis could not be performed since the GWAS for Hcy is not publicly available. Therefore, the effects of the risk of MPDs on plasma homocysteine level should be investigated in the future.

## Conclusions

Our results suggest that altered plasma Hcy levels may be involved in the risk of SCZ or BD-I. However, our findings must be interpreted with caution because of the limitations, such as pleiotropy and population stratification. Future randomized controlled trials are still needed to confirm our two-sample MR findings.

## Data Availability Statement

The original contributions presented in the study are included in the article/Supplementary Material, further inquiries can be directed to the corresponding author/s.

## Author Contributions

JY, XL, and HY designed the study, contributed to analysis and interpretation of data, and wrote the first draft of the manuscript. JY, RX, and QW did the statistical analyses and prepared the tables and figures. XL and HY provided further data interpretation. All authors contributed to drafting the work or revising it critically for important intellectual content and made substantial contributions to the concept and design of the study and acquisition, analysis, and interpretation of data.

## Funding

This study was funded by the National Natural Science Foundation of China (81901358), Natural Science Foundation of Shandong Province (ZR2019BH001 and ZR2021YQ55), Medical and Health Science and Technology Development Plan of Shandong Province (202103090742 and 202103090692), Jining Key Research and Development Program (2020YXNS047 and 2021YXNS077) and Young Taishan Scholars of Shandong Province (tsqn201909146). The funders had no role in the design and conduction of this study.

## Conflict of Interest

The authors declare that the research was conducted in the absence of any commercial or financial relationships that could be construed as a potential conflict of interest.

## Publisher's Note

All claims expressed in this article are solely those of the authors and do not necessarily represent those of their affiliated organizations, or those of the publisher, the editors and the reviewers. Any product that may be evaluated in this article, or claim that may be made by its manufacturer, is not guaranteed or endorsed by the publisher.
