# Supplementary material for: The Effects of Plasma Homocysteine Level on the Risk of Three Major Psychiatric Disorders: A Mendelian Randomization Study
Source: Front Psychiatry. 2022 Mar 21;13:841429. doi: 10.3389/fpsyt.2022.841429 (PMC8977462; doi:10.3389/fpsyt.2022.841429)

# Table S1. Genome-wide significant associated loci for plasma homocysteine level.

| **SNP** | **CHR** | **BP** | **A1** | **A2** | **GENE** | **FRQ** | **BETA** | **SE** | **F** | **P** |
| --- | --- | --- | --- | --- | --- | --- | --- | --- | --- | --- |
| rs12134663 | 1 | 11778589 | A | C | MTHFR | 0.8 | -0.101 | 0.011 | 84.31 | 3.00E-21 |
| rs12780845 | 10 | 17181245 | A | G | CUBN | 0.65 | 0.053 | 0.009 | 34.55 | 8.00E-10 |
| rs12921383 | 16 | 89793345 | T | C | DPEP1 | 0.87 | -0.090 | 0.014 | 41.33 | 8.00E-11 |
| rs154657 | 16 | 89641688 | A | G | DPEP1 | 0.47 | 0.096 | 0.007 | 189.26 | 2.00E-43 |
| rs1801133 | 1 | 11796321 | A | G | MTHFR | 0.34 | 0.158 | 0.007 | 511.41 | 4.00E-104 |
| rs1801222 | 10 | 17114152 | A | G | CUBN | 0.34 | 0.045 | 0.007 | 41.88 | 8.00E-10 |
| rs2251468 | 12 | 120967323 | A | C | HNF1A | 0.65 | -0.051 | 0.007 | 53.50 | 1.00E-12 |
| rs2275565 | 1 | 236885376 | T | G | MTR | 0.21 | -0.054 | 0.009 | 36.27 | 2.00E-10 |
| rs234709 | 21 | 43066854 | T | C | CBS | 0.45 | -0.072 | 0.007 | 105.21 | 4.00E-24 |
| rs2851391 | 21 | 43067294 | T | C | CBS | 0.47 | 0.056 | 0.008 | 49.00 | 2.00E-12 |
| rs42648 | 7 | 90348446 | A | G | GTPB10 | 0.4 | -0.040 | 0.007 | 31.84 | 2.00E-08 |
| rs4660306 | 1 | 45513003 | T | C | MMACHC | 0.33 | 0.044 | 0.007 | 38.62 | 2.00E-09 |
| rs548987 | 6 | 25869143 | C | G | SLC17A3 | 0.13 | 0.060 | 0.01 | 35.64 | 1.00E-08 |
| rs7130284 | 11 | 89415204 | T | C | NOX4 | 0.07 | -0.124 | 0.013 | 91.28 | 2.00E-20 |
| rs7422339 | 2 | 210675783 | A | C | CPS1 | 0.33 | 0.086 | 0.008 | 116.64 | 5.00E-27 |
| rs838133 | 19 | 48756272 | A | G | FUT2 | 0.45 | 0.042 | 0.007 | 36.34 | 7.00E-09 |
| rs9369898 | 6 | 49414480 | A | G | MUT | 0.62 | 0.045 | 0.007 | 41.14 | 2.00E-10 |
| rs957140 | 11 | 89468459 | A | G | NOX4 | 0.45 | -0.045 | 0.008 | 31.64 | 2.00E-08 |

SNP, single nucleotide polymorphism; A1, effect allele; A2, other allele; FRQ, effect allele frequency; SE, standard error;

#

# Table S2. LD correlation matrix for the SNPs used in MR analysis.

|  | **rs12780845** | **rs154657** | **rs1801133** | **rs1801222** | **rs2251468** | **rs2275565** | **rs234709** | **rs42648** | **rs4660306** | **rs548987** | **rs7130284** | **rs838133** | **rs9369898** |
| --- | --- | --- | --- | --- | --- | --- | --- | --- | --- | --- | --- | --- | --- |
| **rs12780845** | 1.000 | -0.007 | -0.083 | -0.013 | -0.043 | 0.014 | -0.054 | -0.045 | 0.042 | 0.014 | 0.024 | -0.080 | -0.048 |
| **rs154657** | -0.007 | 1.000 | 0.048 | -0.062 | 0.023 | 0.067 | -0.042 | -0.010 | 0.000 | -0.047 | 0.045 | -0.002 | -0.078 |
| **rs1801133** | -0.083 | 0.048 | 1.000 | -0.028 | -0.006 | -0.069 | 0.029 | -0.032 | -0.072 | -0.007 | -0.097 | -0.017 | -0.084 |
| **rs1801222** | -0.013 | -0.062 | -0.028 | 1.000 | -0.083 | 0.018 | -0.075 | -0.006 | 0.074 | 0.032 | 0.084 | 0.019 | -0.003 |
| **rs2251468** | -0.043 | 0.023 | -0.006 | -0.083 | 1.000 | 0.006 | 0.007 | -0.074 | 0.028 | 0.091 | -0.026 | -0.058 | -0.062 |
| **rs2275565** | 0.014 | 0.067 | -0.069 | 0.018 | 0.006 | 1.000 | -0.029 | -0.028 | -0.032 | 0.009 | 0.014 | -0.001 | -0.037 |
| **rs234709** | -0.054 | -0.042 | 0.029 | -0.075 | 0.007 | -0.029 | 1.000 | -0.057 | -0.031 | -0.078 | -0.069 | 0.018 | -0.018 |
| **rs42648** | -0.045 | -0.010 | -0.032 | -0.006 | -0.074 | -0.028 | -0.057 | 1.000 | 0.059 | -0.031 | 0.042 | -0.018 | 0.064 |
| **rs4660306** | 0.042 | 0.000 | -0.072 | 0.074 | 0.028 | -0.032 | -0.031 | 0.059 | 1.000 | 0.022 | -0.030 | -0.044 | -0.006 |
| **rs548987** | 0.014 | -0.047 | -0.007 | 0.032 | 0.091 | 0.009 | -0.078 | -0.031 | 0.022 | 1.000 | 0.005 | -0.021 | 0.067 |
| **rs7130284** | 0.024 | 0.045 | -0.097 | 0.084 | -0.026 | 0.014 | -0.069 | 0.042 | -0.030 | 0.005 | 1.000 | 0.019 | 0.024 |
| **rs838133** | -0.080 | -0.002 | -0.017 | 0.019 | -0.058 | -0.001 | 0.018 | -0.018 | -0.044 | -0.021 | 0.019 | 1.000 | -0.047 |
| **rs9369898** | -0.048 | -0.078 | -0.084 | -0.003 | -0.062 | -0.037 | -0.018 | 0.064 | -0.006 | 0.067 | 0.024 | -0.047 | 1.000 |

# Table S3. The genetic instruments for Mendelian Randomization analysis of homocysteine (exposure) and SCZ (outcome)

| **SNP** | **A1** | **A2** | **FRQ** | **bzx** | **bzx_se** | **bzx_pval** | **bzy** | **bzy_se** | **bzy_pval** |
| --- | --- | --- | --- | --- | --- | --- | --- | --- | --- |
| rs12780845 | A | G | 0.65 | 0.053 | 0.009 | 8.00E-10 | -0.017 | 0.008 | 4.31E-02 |
| rs154657 | A | G | 0.47 | 0.096 | 0.007 | 2.00E-43 | 0.040 | 0.009 | 4.05E-06 |
| rs1801133 | A | G | 0.34 | 0.158 | 0.007 | 4.00E-104 | 0.007 | 0.008 | 3.88E-01 |
| rs1801222 | A | G | 0.34 | 0.045 | 0.007 | 8.00E-10 | 0.018 | 0.009 | 3.58E-02 |
| rs2251468 | A | C | 0.65 | -0.051 | 0.007 | 1.00E-12 | -0.036 | 0.008 | 5.93E-06 |
| rs2275565 | T | G | 0.21 | -0.054 | 0.009 | 2.00E-10 | 0.003 | 0.010 | 7.43E-01 |
| rs234709 | T | C | 0.45 | -0.072 | 0.007 | 4.00E-24 | -0.020 | 0.008 | 1.69E-02 |
| rs42648 | A | G | 0.4 | -0.040 | 0.007 | 2.00E-08 | 0.014 | 0.008 | 7.89E-02 |
| rs4660306 | T | C | 0.33 | 0.044 | 0.007 | 2.00E-09 | 0.004 | 0.009 | 6.57E-01 |
| rs548987 | C | G | 0.13 | 0.060 | 0.01 | 1.00E-08 | -0.079 | 0.012 | 1.64E-11 |
| rs7130284 | T | C | 0.07 | -0.124 | 0.013 | 2.00E-20 | -0.007 | 0.013 | 5.98E-01 |
| rs838133 | A | G | 0.45 | 0.042 | 0.007 | 7.00E-09 | 0.042 | 0.009 | 4.67E-06 |
| rs9369898 | A | G | 0.62 | 0.045 | 0.007 | 2.00E-10 | 0.003 | 0.008 | 6.56E-01 |

SNP, single nucleotide polymorphism; A1, effect allele; A2, other allele; FRQ, effect allele frequency; bzx, the effect size of a1 on exposure; bzx_se, standard error of bzx; bzx_pval, p value for bzx; bzy, the effect size of a1 on outcome; bzy_se, standard error of bzy; bzy_pval, p value for bzy.

# Table S3. The genetic instruments for Mendelian Randomization analysis of homocysteine (exposure) and MDD (outcome)

| **SNP** | **A1** | **A2** | **FRQ** | **bzx** | **bzx_se** | **bzx_pval** | **bzy** | **bzy_se** | **bzy_pval** |
| --- | --- | --- | --- | --- | --- | --- | --- | --- | --- |
| rs12780845 | A | G | 0.65 | 0.053 | 0.009 | 8.00E-10 | -0.0005 | 0.008 | 9.57E-01 |
| rs154657 | A | G | 0.47 | 0.096 | 0.007 | 2.00E-43 | -0.0006 | 0.008 | 9.39E-01 |
| rs1801133 | A | G | 0.34 | 0.158 | 0.007 | 4.00E-104 | -0.0065 | 0.009 | 4.54E-01 |
| rs1801222 | A | G | 0.34 | 0.045 | 0.007 | 8.00E-10 | -0.0135 | 0.008 | 1.00E-01 |
| rs2251468 | A | C | 0.65 | -0.051 | 0.007 | 1.00E-12 | 0.003404 | 0.008 | 6.84E-01 |
| rs2275565 | T | G | 0.21 | -0.054 | 0.009 | 2.00E-10 | 0.001 | 0.010 | 9.20E-01 |
| rs234709 | T | C | 0.45 | -0.072 | 0.007 | 4.00E-24 | -0.0033 | 0.008 | 6.92E-01 |
| rs42648 | A | G | 0.4 | -0.040 | 0.007 | 2.00E-08 | -0.0049 | 0.008 | 5.48E-01 |
| rs4660306 | T | C | 0.33 | 0.044 | 0.007 | 2.00E-09 | -0.0108 | 0.008 | 1.93E-01 |
| rs548987 | C | G | 0.13 | 0.060 | 0.01 | 1.00E-08 | -0.0311 | 0.012 | 8.25E-03 |
| rs7130284 | T | C | 0.07 | -0.124 | 0.013 | 2.00E-20 | 0.018704 | 0.015 | 2.08E-01 |
| rs838133 | A | G | 0.45 | 0.042 | 0.007 | 7.00E-09 | -0.0044 | 0.009 | 6.06E-01 |
| rs9369898 | A | G | 0.62 | 0.045 | 0.007 | 2.00E-10 | 0.010703 | 0.008 | 1.90E-01 |

SNP, single nucleotide polymorphism; A1, effect allele; A2, other allele; FRQ, effect allele frequency; bzx, the effect size of a1 on exposure; bzx_se, standard error of bzx; bzx_pval, p value for bzx; bzy, the effect size of a1 on outcome; bzy_se, standard error of bzy; bzy_pval, p value for bzy.Table S5. The genetic instruments for Mendelian Randomization analysis of homocysteine (exposure) and BD (outcome)

| **SNP** | **A1** | **A2** | **FRQ** | **bzx** | **bzx_se** | **bzx_pval** | **bzy** | **bzy_se** | **bzy_pval** |
| --- | --- | --- | --- | --- | --- | --- | --- | --- | --- |
| rs12780845 | A | G | 0.65 | 0.0529 | 0.009 | 8.00E-10 | -0.016 | 0.010 | 1.05E-01 |
| rs154657 | A | G | 0.47 | 0.0963 | 0.007 | 2.00E-43 | 0.023 | 0.010 | 1.39E-02 |
| rs1801133 | A | G | 0.34 | 0.1583 | 0.007 | 4.00E-104 | 0.019 | 0.010 | 6.30E-02 |
| rs1801222 | A | G | 0.34 | 0.0453 | 0.007 | 8.00E-10 | 0.008 | 0.010 | 4.49E-01 |
| rs2251468 | A | C | 0.65 | -0.0512 | 0.007 | 1.00E-12 | -0.040 | 0.010 | 9.39E-05 |
| rs2275565 | T | G | 0.21 | -0.0542 | 0.009 | 2.00E-10 | -0.007 | 0.011 | 5.35E-01 |
| rs234709 | T | C | 0.45 | -0.0718 | 0.007 | 4.00E-24 | -0.010 | 0.010 | 3.15E-01 |
| rs42648 | A | G | 0.4 | -0.0395 | 0.007 | 2.00E-08 | 0.009 | 0.010 | 3.31E-01 |
| rs4660306 | T | C | 0.33 | 0.0435 | 0.007 | 2.00E-09 | -0.019 | 0.010 | 4.92E-02 |
| rs548987 | C | G | 0.13 | 0.0597 | 0.01 | 1.00E-08 | -0.046 | 0.014 | 1.28E-03 |
| rs7130284 | T | C | 0.07 | -0.1242 | 0.013 | 2.00E-20 | 0.025 | 0.017 | 1.39E-01 |
| rs838133 | A | G | 0.45 | 0.0422 | 0.007 | 7.00E-09 | 0.009 | 0.010 | 3.81E-01 |
| rs9369898 | A | G | 0.62 | 0.0449 | 0.007 | 2.00E-10 | 0.015 | 0.010 | 1.12E-01 |

SNP, single nucleotide polymorphism; A1, effect allele; A2, other allele; FRQ, effect allele frequency; bzx, the effect size of a1 on exposure; bzx_se, standard error of bzx; bzx_pval, p value for bzx; bzy, the effect size of a1 on outcome; bzy_se, standard error of bzy; bzy_pval, p value for bzy.

Table S5. The genetic instruments for Mendelian Randomization analysis of homocysteine (exposure) and BD-I (outcome)

| **SNP** | **A1** | **A2** | **FRQ** | **bzx** | **bzx_se** | **bzx_pval** | **bzy** | **bzy_se** | **bzy_pval** |
| --- | --- | --- | --- | --- | --- | --- | --- | --- | --- |
| rs12780845 | A | G | 0.65 | 0.0529 | 0.009 | 8.00E-10 | -0.017 | 0.0124 | 1.68E-01 |
| rs154657 | A | G | 0.47 | 0.0963 | 0.007 | 2.00E-43 | 0.035 | 0.0116 | 2.60E-03 |
| rs1801133 | A | G | 0.34 | 0.1583 | 0.007 | 4.00E-104 | 0.025 | 0.0122 | 4.12E-02 |
| rs1801222 | A | G | 0.34 | 0.0453 | 0.007 | 8.00E-10 | 0.007 | 0.0124 | 5.50E-01 |
| rs2251468 | A | C | 0.65 | -0.0512 | 0.007 | 1.00E-12 | -0.031 | 0.012 | 9.07E-03 |
| rs2275565 | T | G | 0.21 | -0.0542 | 0.009 | 2.00E-10 | -0.008 | 0.014 | 5.47E-01 |
| rs234709 | T | C | 0.45 | -0.0718 | 0.007 | 4.00E-24 | -0.004 | 0.0118 | 7.20E-01 |
| rs42648 | A | G | 0.4 | -0.0395 | 0.007 | 2.00E-08 | 0.010 | 0.0118 | 4.00E-01 |
| rs4660306 | T | C | 0.33 | 0.0435 | 0.007 | 2.00E-09 | -0.026 | 0.0122 | 3.02E-02 |
| rs548987 | C | G | 0.13 | 0.0597 | 0.01 | 1.00E-08 | -0.038 | 0.0177 | 2.98E-02 |
| rs7130284 | T | C | 0.07 | -0.1242 | 0.013 | 2.00E-20 | 0.019 | 0.0214 | 3.66E-01 |
| rs838133 | A | G | 0.45 | 0.0422 | 0.007 | 7.00E-09 | 0.012 | 0.0124 | 3.20E-01 |
| rs9369898 | A | G | 0.62 | 0.0449 | 0.007 | 2.00E-10 | 0.032 | 0.0119 | 6.69E-03 |

SNP, single nucleotide polymorphism; A1, effect allele; A2, other allele; FRQ, effect allele frequency; bzx, the effect size of a1 on exposure; bzx_se, standard error of bzx; bzx_pval, p value for bzx; bzy, the effect size of a1 on outcome; bzy_se, standard error of bzy; bzy_pval, p value for bzy.

Table S5. The genetic instruments for Mendelian Randomization analysis of homocysteine (exposure) and BD-II (outcome)

| **SNP** | **A1** | **A2** | **FRQ** | **bzx** | **bzx_se** | **bzx_pval** | **bzy** | **bzy_se** | **bzy_pval** |
| --- | --- | --- | --- | --- | --- | --- | --- | --- | --- |
| rs12780845 | A | G | 0.65 | 0.0529 | 0.009 | 8.00E-10 | -0.051 | 0.021 | 1.58E-02 |
| rs154657 | A | G | 0.47 | 0.0963 | 0.007 | 2.00E-43 | -0.026 | 0.0199 | 2.00E-01 |
| rs1801133 | A | G | 0.34 | 0.1583 | 0.007 | 4.00E-104 | 0.028 | 0.0209 | 1.81E-01 |
| rs1801222 | A | G | 0.34 | 0.0453 | 0.007 | 8.00E-10 | 0.004 | 0.0206 | 8.47E-01 |
| rs2251468 | A | C | 0.65 | -0.0512 | 0.007 | 1.00E-12 | -0.035 | 0.0204 | 8.49E-02 |
| rs2275565 | T | G | 0.21 | -0.0542 | 0.009 | 2.00E-10 | 0.017 | 0.0235 | 4.62E-01 |
| rs234709 | T | C | 0.45 | -0.0718 | 0.007 | 4.00E-24 | -0.004 | 0.0203 | 8.47E-01 |
| rs42648 | A | G | 0.4 | -0.0395 | 0.007 | 2.00E-08 | 0.013 | 0.02 | 4.99E-01 |
| rs4660306 | T | C | 0.33 | 0.0435 | 0.007 | 2.00E-09 | -0.013 | 0.0205 | 5.29E-01 |
| rs548987 | C | G | 0.13 | 0.0597 | 0.01 | 1.00E-08 | -0.075 | 0.0305 | 1.38E-02 |
| rs7130284 | T | C | 0.07 | -0.1242 | 0.013 | 2.00E-20 | -0.020 | 0.0369 | 5.89E-01 |
| rs838133 | A | G | 0.45 | 0.0422 | 0.007 | 7.00E-09 | 0.011 | 0.0205 | 6.11E-01 |
| rs9369898 | A | G | 0.62 | 0.0449 | 0.007 | 2.00E-10 | -0.005 | 0.0202 | 8.15E-01 |

SNP, single nucleotide polymorphism; A1, effect allele; A2, other allele; FRQ, effect allele frequency; bzx, the effect size of a1 on exposure; bzx_se, standard error of bzx; bzx_pval, p value for bzx; bzy, the effect size of a1 on outcome; bzy_se, standard error of bzy; bzy_pval, p value for bzy.

**Supplementary Figure 1. Leave-one-out sensitivity analysis for** homocysteine level and schizophrenia**.** The solid lines represent 95% confidence intervals. Hcy, homocysteine; SCZ, schizophrenia; SNP, single nucleotide polymorphism.


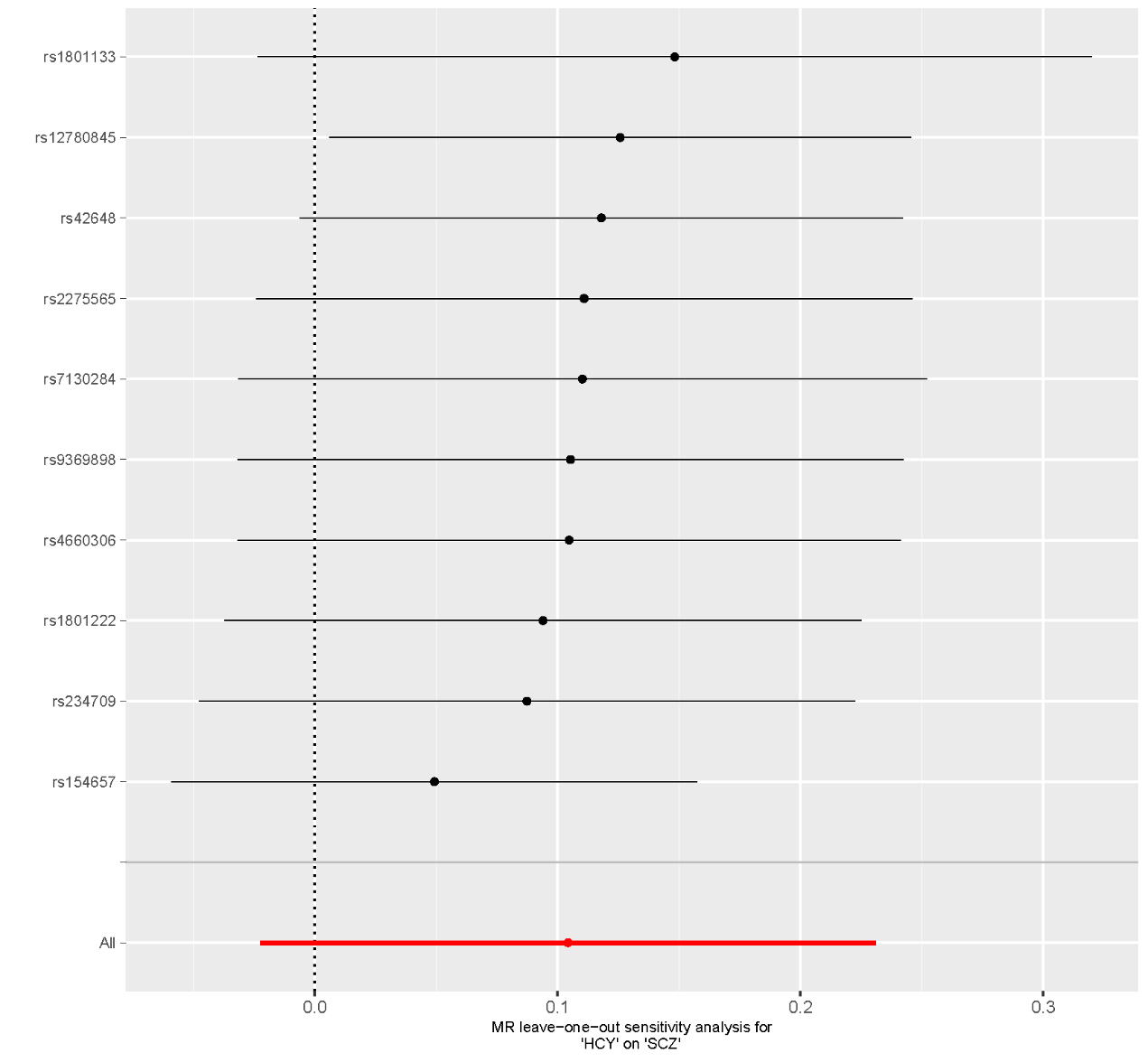


**Supplementary Figure 2. Leave-one-out sensitivity analysis for** homocysteine level and major depressive disorder**.** The solid lines represent 95% confidence intervals. Hcy, homocysteine; MDD, major depressive disorder; SNP, single nucleotide polymorphism.


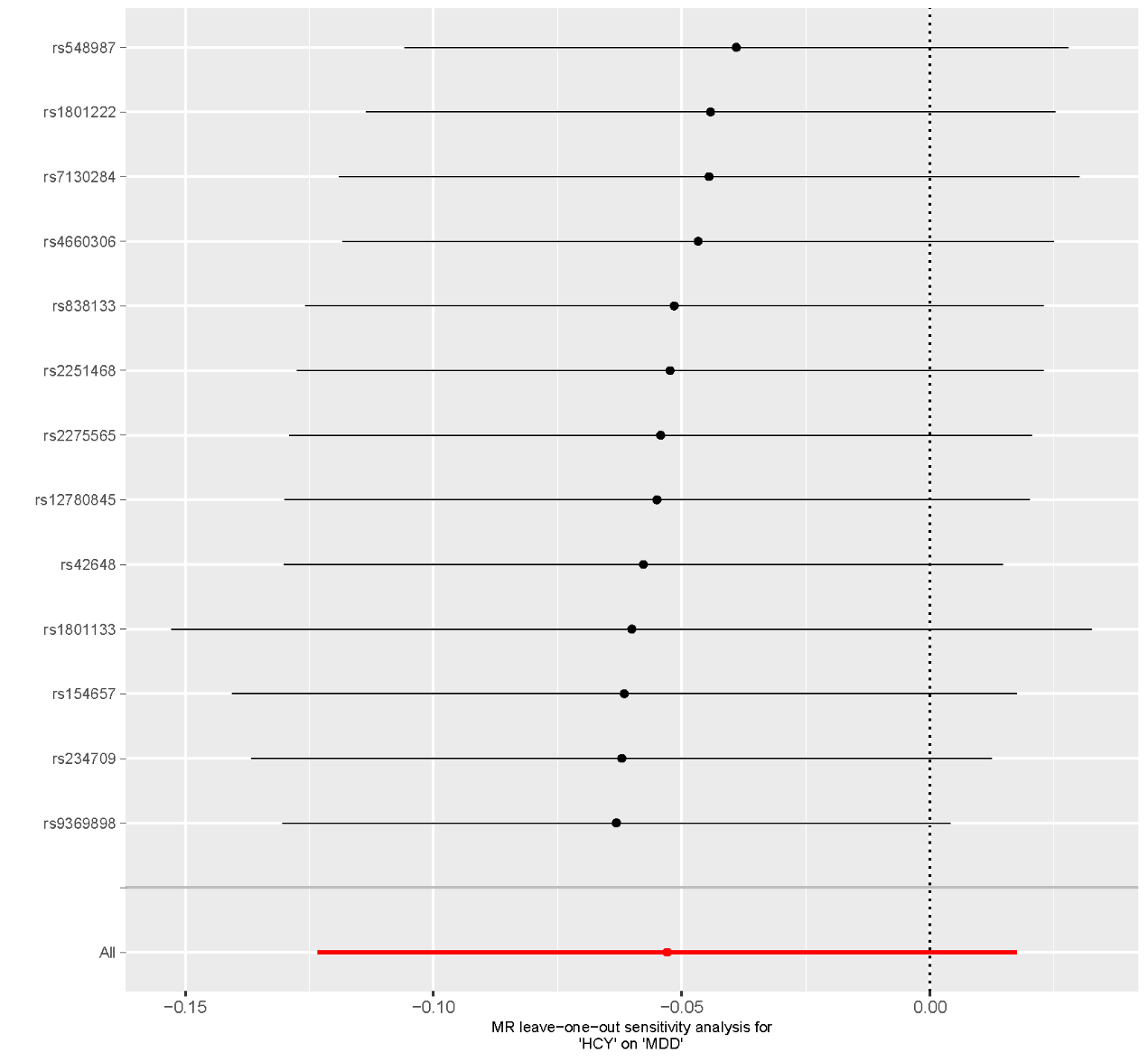


**Supplementary Figure 3. Leave-one-out sensitivity analysis for** homocysteine level and bipolar disorder**.** The solid lines represent 95% confidence intervals. Hcy, homocysteine; BD, bipolar disorder; SNP, single nucleotide polymorphism.


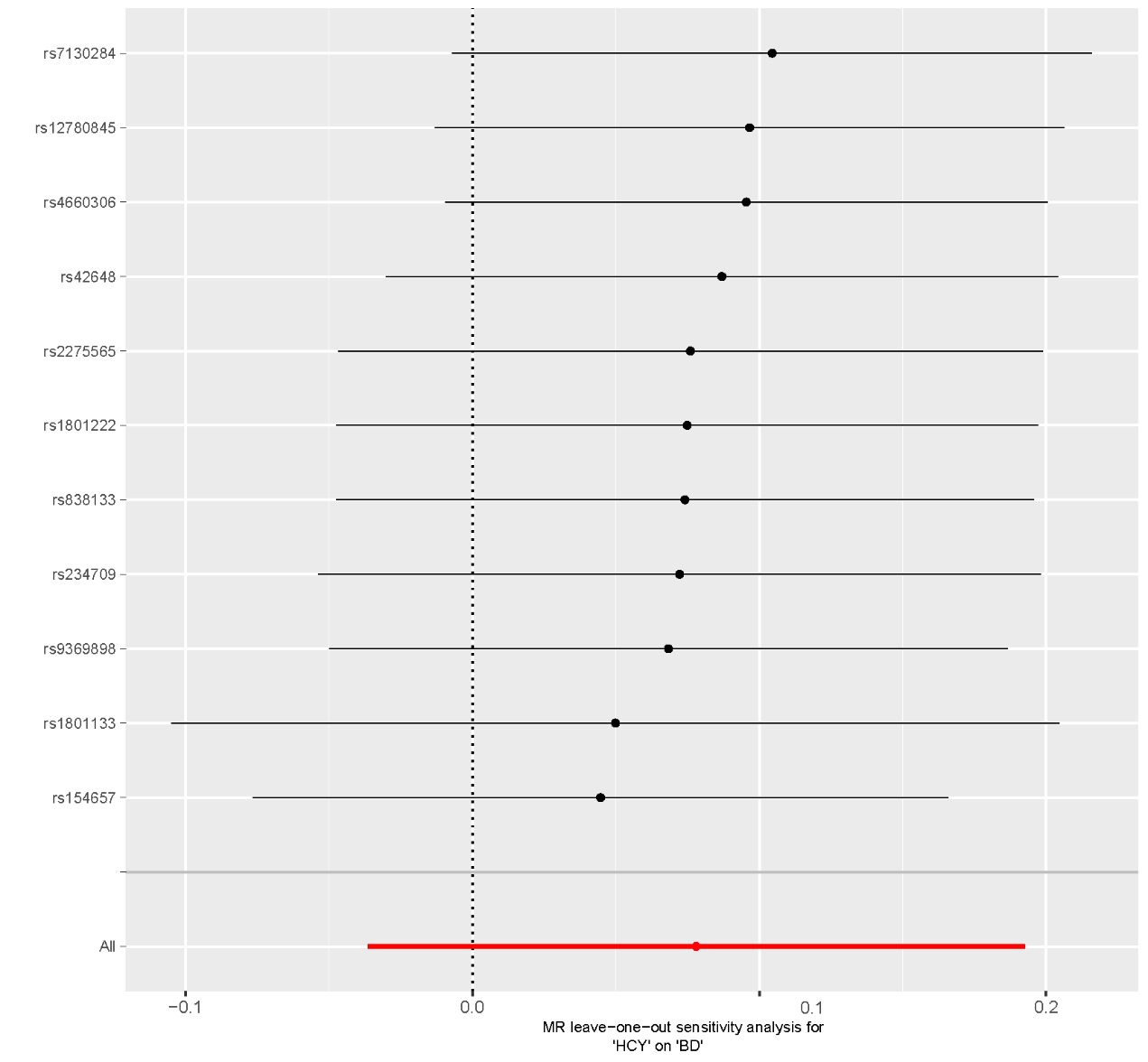


**Supplementary Figure 4. Leave-one-out sensitivity analysis for** homocysteine level and bipolar disorder-I**.** The solid lines represent 95% confidence intervals. Hcy, homocysteine; BD-I, bipolar disorder I type; SNP, single nucleotide polymorphism.


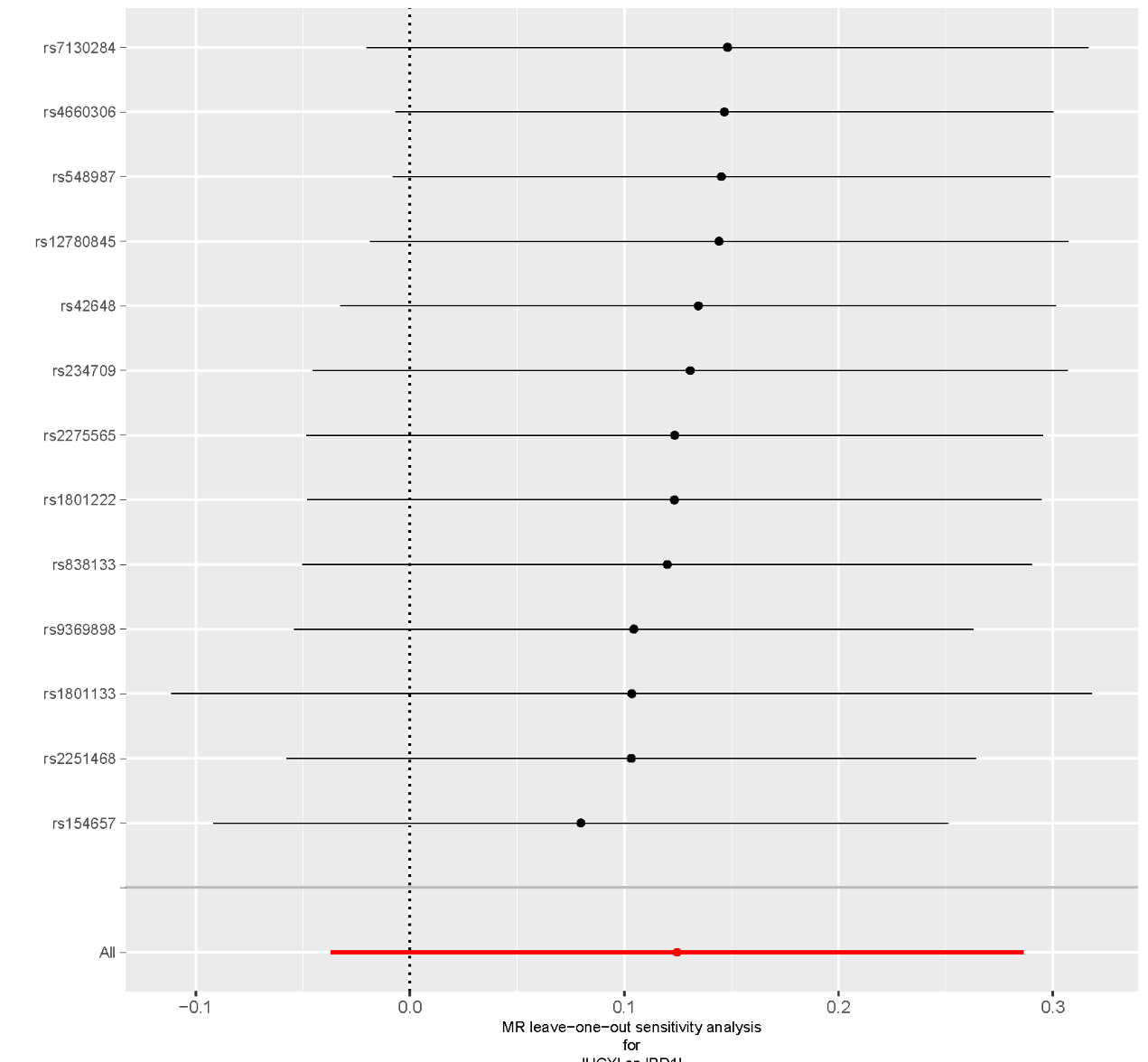


**Supplementary Figure 5. Leave-one-out sensitivity analysis for** homocysteine level and bipolar disorder-II**.** The solid lines represent 95% confidence intervals. Hcy, homocysteine; BD-II, bipolar disorder I type; SNP, single nucleotide polymorphism.


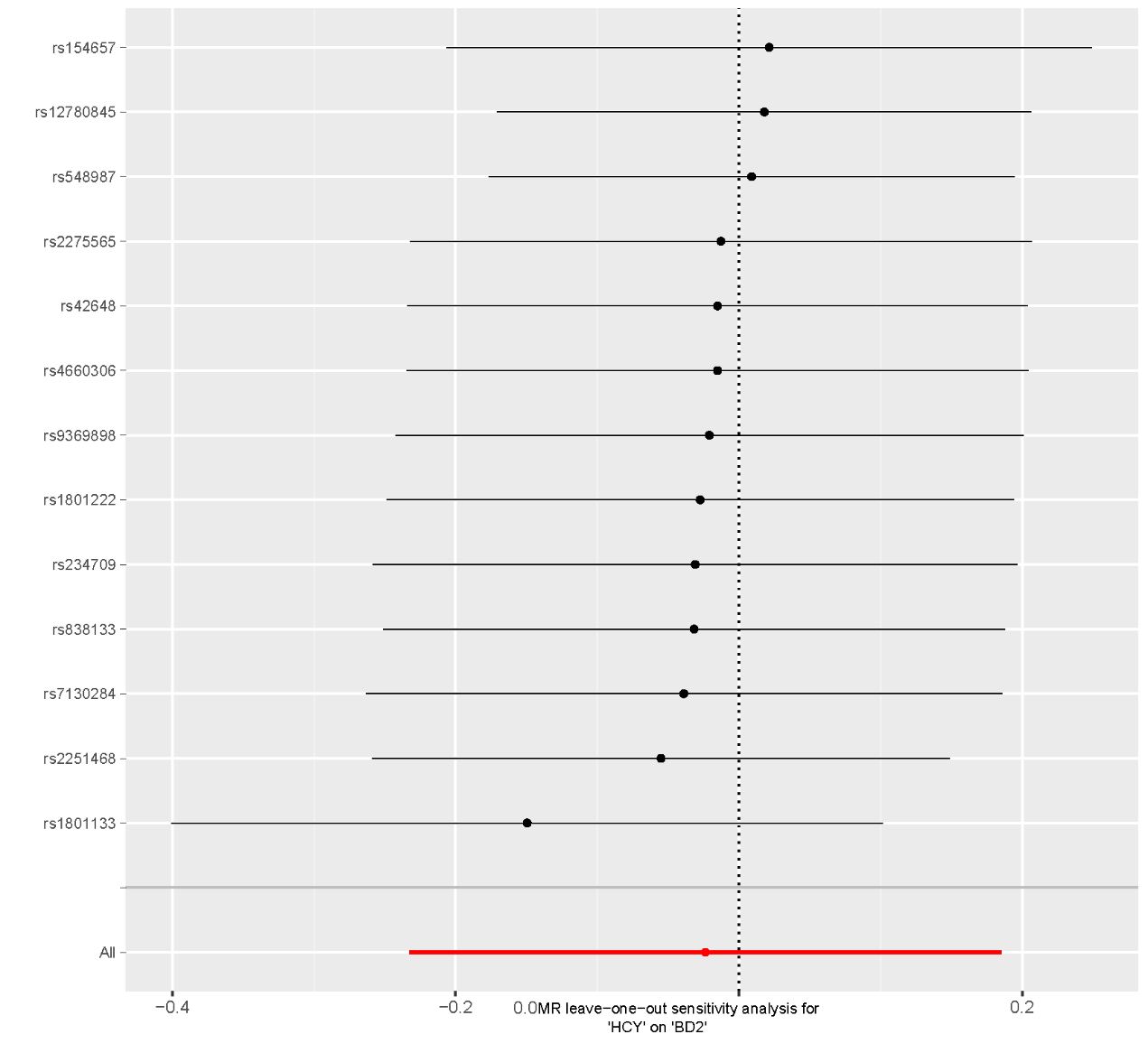

Supplement: Supplementary file 1 [file Data_Sheet_1.docx]
